# Society Meetings

**Published:** 1915-06

**Authors:** 


### SOCIETY MEETINGS

---

Brief reports and announcements of meetings of societies of Western New York, and those of general scope, are requested from Secretaries. Copy should be on hand the fifteenth of the month. Full transactions will be published at cost of composition.

---

**DOCTOR: Is your Society properly represented here? If not, please bring our standing announcement to the attention of the members.**

---

**The Seventh Pan-American Congress** will meet in San Francisco, June 17th-21st inclusive. It assembles pursuant to accordance with an act of Congress approved March 3, 1915.

The Congress will meet in seven sections, viz: (1) Medicine; (2) Surgery; (3) Obstetrics and Gynecology; (4) Anatomy, Physiology, Pathology and Bacteriology; (5) Tropical Medicine and General Sanitation; (6) Laryngology; Rhinology and Otology; (7) Medical Literature.

All members of the organized medical profession of the constituent countries are eligible. Membership fee $5.00 entitling to a complete set of the transactions. Advance registrations are solicited and should be sent with fee to the Treasurer, Dr. Henry P. Newman, Timken Building, San Diego, California.

The general railroad rate of one fare for the round trip, good for three months, made on account of the Panama-Pacific Exposition at San Francisco, and the California Exposition at San Diego is available for the Pan-American Medical Congress.

---

**The Women's Medical Society of New York State** met in Buffalo April 26, before the meeting of the State Society. The following officers were elected: President, Dr. Maud J. Frye of Buffalo; Vice-presidents, Dr. Eveline P. Ballantyne of Rochester, Dr. Annie S. Daniel of N. Y., Dr. Mary S. Macy of N. Y.; Secretary, Dr. Mary Gage Day of Kingston; Treasurer, Dr. Florence McKay of Rochester. Dr. Angenette Parry of N. Y. acted as toast-mistress at the banquet.

---

**The Alumni Association,** Medical Dept., University of Buffalo, will hold its 40th annual meeting June 1-4. Coming just at the time of the appearance of this issue, it is not worth while to attempt an advance notice but we expect to publish the transactions in the July issue.

---

**The Buffalo Academy of Medicine.** Section on Surgery, May 5. The Law and Psychology of Collections, Charles B. Dress, Jr., Esq.

May 12. Medical Section, Diet and Nutrition in Health, Dr. A. P. Sy; The Problems of Nutrition in Disease, Dr. J. L. Butsch.

---

**Medical Society of the County of Monroe,** Tuesday, May 18, 1915. Clinics.

1. General Hospital at 10—Medicine—R. Moore in the Medical Wards.
2. General Hospital at 10—Surgery—H. T. Williams in the Operating Rooms.
3. General Hospital at 10—Obstetrics—J. K. Quigley in the Hart Memorial Building.
4. General Hospital at 10—Orthopedics—H. L. Prince on the Fourth Floor, West Hall.
5. St. Mary's Hospital at 10—Surgery—O. E. Jones in the Operating Rooms.
6. St. Mary's Hospital at 10—Throat and Nose—G. G. Carroll in the Operating Rooms.
7. Children's Hospital, Rochester Public Health Association at 10—E. G. Whipple, on the Examination of the Chest.

Scientific Program: Impacted Fracture of Hip, C. S. Starr. Injuries to the Knee Joint, H. L. Prince. A Rare Osseous Tumor of the Leg, F. W. Seymour. A Method of finding Tubercle Bacilli in Spinal Fluid, J. Roby. The Diagnosis of Early Pulmonary Tuberculosis in General Practice, E. G. Whipple. Tobacco—The Effect of Smoking on the Circulation, J. Aikman. Diabetes Mellitus, J. R. Williams. Roentgen Examination of the Stomach and Duodenum, (Lantern Slides), M. B. Palmer. Some Points in the Technic of Caesarean Section, (Lantern Slides), W. M. Brown. Some Aspects of Modern Obstetrics, J. K. Quigley. Construction of an Artificial Vagina, W. D. Ward. Blood in the Urine, T. G. Touse. Plans for a more efficient County Society, C. G. Lenhart, Spencerport.

---

**Medical Meetings at the Panama-Pacific Exposition.** (Lowell Otes Reese). Pac. Med. Jour.

Pacific Coast Oto-Ophthalmological Society, Sessions June 14, 15, 16.

American Society of Tropical Medicine, Sessions June 14, 15, 16.

American Association Medical Milk Commissions, Sessions June 17, 18, 19.

Pan-American Medical Congress, Sessions June 17, 18, 19.

American Climatological and Clinical Association, Sessions June 18, 19.

American College of Surgeons, Session June 21.

Medical Society of the State of California, Session June 21.

American Proctologic Society, Sessions June 21, 22.

American Therapeutic Society, Sessions June 21, 22.

American Hospital Association, Sessions June 21, 22, 23, 24, 25.

American Medical Association, June 21, 22, 23, 24, 25, 26.

American School Hygiene Association, June 25, 26.

American Academy of Medicine, June 25, 26, 27, 28.

The Medical Society of the Canal Zone probably will hold its sessions in June also; the Pacific Coast Association of Railway Surgeons, later probably in September, and the American Medical Veterinary Association in August or September.

The Panama-Pacific Dental Congress, International Dental Association, National Association of Dental Examiners, Dental Faculties' Association of American Universities, International Dental Federation, California State Dental Association, Southern California Dental Association, Utah State Dental Society, California Pharmaceutical Association, American Pharmaceutical Faculties, California Nurses' Association, American Nurses' Association, and National Organization for Public Health Nursing. will also meet in San Francisco during the exposition.

The exposition Auditorium, itself one of the memorable features of the Panama-Pacific International Exposition and a permanent monument to America's achievements, lends itself admirably to the requirements of the medical period. It contains 21 convention halls, the smallest having a seating capacity of 200 and the largest 11,600. In addition there are 19 rooms for committee and section meetings, with a seating capacity of from 30 to 125 each. Centrally located, upon the city's main thoroughfare, which, by the way, is one of the few great gateway thoroughfares of the world's travel, the Auditorium is practically at the doors of all the hotels, and is easily accessible from the exposition grounds. The building is abundantly supplied with lockers, lavatories, checking spaces, telephones, elevators, call bells and excellent heat and ventilation. In the wide main hall of the Auditorium there will be commercial and scientific exhibits of great interest.

---

At the meeting of the **Elmira Clinical Society** on May 24th, Dr. C. A. Bentz of Buffalo read a paper on "Autopsy Findings in Cases of Sudden Death." Dr. Hamilton of Elmira, "Curbstone Prescribing."

---

**Rochester Pathological Society,** May 6, Cystitis, T. G. Tousey.

May 20, Review of the Surgical Treatment of Hydrocephalus, by Irving S. Haynes of New York.

June 3, Acute Osteomgelitis and its surgical treatment, Joseph W. Magill of Rochester.

June 17, Annual Meeting and Picnic.

**Blackwell Medical Society,** Rochester.
May 13, Ether Anaesthesia, Elizabeth A. Merle.
June 19, President's Address. Annual Meeting.
**Rochester Academy of Medicine.**
May 12, Bautis Disease, J. R. Williams; Splenectomy, F. W. Zimmer; Blood Changes, C. O. Boswell.

On May 13, **The Medical and Surgical League** held their annual dinner at McGerald's Cafe. Rabbi Max Drob gave the address of the evening, his subject being, "The Jewish Attitude Toward Medical Science." The following officers were elected: Dr. Leonard Reu, President; Dr. Arthur C. Schaefer, Vice-president; Dr. Frank C. Kleckner, Secretary; Dr. Edmund P. Reimann, Treasurer; Board of Governors, Dr. George W. Seitz, Dr. Julius Richter, Dr. Augustus W. Hengerer, Dr. Robert S. Taylor, Dr. Francis M. O'Gorman.

